# Focal segmental glomerulosclerosis ACTN4 mutants binding to actin: regulation by phosphomimetic mutations

**DOI:** 10.1038/s41598-019-51825-2

**Published:** 2019-10-29

**Authors:** Hanshuang Shao, Bentley Wingert, Astrid Weins, Martin R. Pollak, Carlos Camacho, Alan Wells

**Affiliations:** 1 Departments of Pathology, University of Pittsburgh, Pittsburgh, PA 15213 USA; 2 Departments of Computational and Systems Biology, University of Pittsburgh, Pittsburgh, PA 15213 USA; 3 Pittsburgh VA Health System, Pittsburgh, PA 15213 USA; 4 Renal and Translational Medicine Divisions, Department of Medicine, Brigham and Women’s Hospital and Harvard Medical School, Boston, MA 02115 USA; 5 Harvard Medical School, Beth Israel Deaconess Medical Center, Boston, MA 02215 USA; 6 Division of Nephrology, Department of Medicine, Beth Israel Deaconess Medical Center, Boston, MA 02215 USA

**Keywords:** Cell adhesion, Cell migration, Cytoskeleton

## Abstract

Natural mutations such as lysine 255 to glutamic acid (K to E), threonine 259 to isoleucine (T to I) and serine 262 to proline (S to P) that occur within the actin binding domain of alpha-actinin-4 (ACTN4) cause an autosomal dominant form of focal segmental glomerulosclerosis (FSGS) in affected humans. This appears due to elevated actin binding propensity in podocytes resulting in a ‘frozen’ cytoskeleton. What is challenging is how this cellular behavior would be compatible with other cell functions that rely on cytoskeleton plasticity. Our previous finding revealed that wild type ACTN4 can be phosphorylated at tyrosine 4 and 31 upon stimulation by epidermal growth factor (EGF) to reduce the binding to actin cytoskeleton. We queried whether the elevated actin binding activity of FSGS mutants can be downregulated by EGF-mediated phosphorylation, to discern a mechanism by which the actin-cytoskeleton can be released in FSGS. In this manuscript, we first constructed variants with Y4/31E to mimic the phosphorylation at tyrosines 4 and 31 based on earlier modeling simulations that predicted that this would bury the actin binding domains and lead to a decrease in actin binding activity. We found that Y4/31E significantly reduced the actin binding activity of K255E, T259I and S262P, dramatically preventing them from aggregating in, and inhibiting motility of, podocytes, fibroblasts and melanoma cells. A putative kinase target site at Y265 in the actin binding domain was also generated as a phosphomimetic ACTN4 Y265E that demonstrated even greater binding to actin filaments than K255E and the other FSGS mutants. That the tyrosine kinase regulation of FSGS mutation binding to actin filaments can occur in cells was shown by phosphorylation on Y4 and Y31 of the K225E after extended exposure of cells to EGF, with a decrease in ACTN4 aggregates in fibroblasts. These findings will provide evidence for targeting the N-termini of FSGS ACTN4 mutants to downregulate their actin binding activities for ameliorating the glomerulosclerotic phenotype of patients.

## Introduction

The alpha-actinin (ACTN) family of F-actin crosslinking proteins consists of four members which are highly homologous in amino acids and divided into two groups based on their expression in either muscle cell or ubiquitously in other non-muscle cells. The non-muscle family members, ACTN1 and ACTN4 have been implicated in F-actin crosslinking that modules cell interactions with the substratum and during motility^1–3^. A potential rationale for the seemingly redundant ACTN4 arose when this molecule was not only found to bridge the cytoskeleton to the membrane during cell migration^4,5^, but also by a unique but highly conserved dual tyrosine-directed phosphorylation in the amino terminal intrinsically disordered region^6^. The further relevance of this isoform came to the fore when Pollak and colleagues found that patients with focal segmental glomerulosclerosis (FSGS), an autosomal dominant disease in which the podocytes appear to be ‘overly’ attached to the substratum, comprised a series of mutations occurring in ACTN4^7,8^. Among mutations that all occur within the conserved actin binding domain (ABD) of ACTN4, a variant K255E presents most enhanced actin binding activity *in vitro* and forms aggregates in cells; the other mutations including T259I and S262P also bind actin more strongly compared to wild type.

The question as to why the disease phenotype is restricted to a unique cell population was answered when immunoblotting data revealed that human kidney expresses high levels of ACTN4 but not ACTN1, and ACTN4 is most prominently presented in podocytes of all the cell types in the kidney^7,9–11^. In the past decade, a number of studies had been focused on the mechanism by which how ACTN4 variants with increased actin binding activity could cause the noted disease. For example, K255E mutant had been generally thought to impair the filtration function of kidney through decreasing the dynamic of the podocyte-determined glomerular pores by freezing the cytoskeleton due to the formation of aggregates of K255E and actin filaments^12–14^. These findings brought up the further question of how the cells could then turnover the actin cytoskeleton when needed.

Structurally, ACTN4, similar to other alpha-actinins, consists of a long rod domain that connects the amino terminal actin binding domain (ABD) and the carboxyl calcium binding motif (CaM) and presents in antiparallel homodimers. The ABD contains the cleft that binds to actin filaments^2,5,15–17^. All alpha-actinins contain an unstructured amino-terminal string of amino acids. However, the first 19 amino acids of ACTN4 are absent in ACTN1. Uniquely, but conserved at least from teleost fish^6^, ACTN4 presents two tyrosines that are phosphorylated in a hierarchical manner to dramatically decrease binding to actin filaments^15^. We previously found that growth factors led to ACTN4 phosphorylation first on tyrosine 4, that exposed the second site of phosphorylation on tyrosine 31^6,18,19^. This provides a mechanism by which binding of ACTN4 to actin can be modulated.

Herein, we tested whether the actin binding of FSGS ACTN4 mutants could be regulated similarly to WT ACTN4, via targeting the intrinsically disordered amino terminus. Indeed, we found that introducing a Y4/31E phosphomimetic mutation significantly decreased actin binding activity of all K255E, T259I and S262P ACTN4 and prevented the aggregations of these mutants in cells. The limited cell migration in cells carrying these FSGS mutant ACTN4 can be efficiently rescued by introducing the phosphomimetic mutations. This provides a proof of principle but is not physiological. Thus, more importantly, continuous EGF stimulation of cells in which nascent K255E-eGFP proteins are translated and exported into cytoplasm results in a significant increase in the tyrosyl phosphorylation of the ACTN4 carrying K255E followed, by a disappearance of aggregates and a more physiological cellular distribution of the ACTN4. Our findings imply that the impaired functions of these mutants can be regulated physiologically and suggest approaches to alleviating the cellular pathophysiology of FSGS.

## Results

### Phosphomimetic mutations of K255E, T259I and S262P ACTN4 on Y4/31 decrease their F-actin binding activities

**Figure 1:**
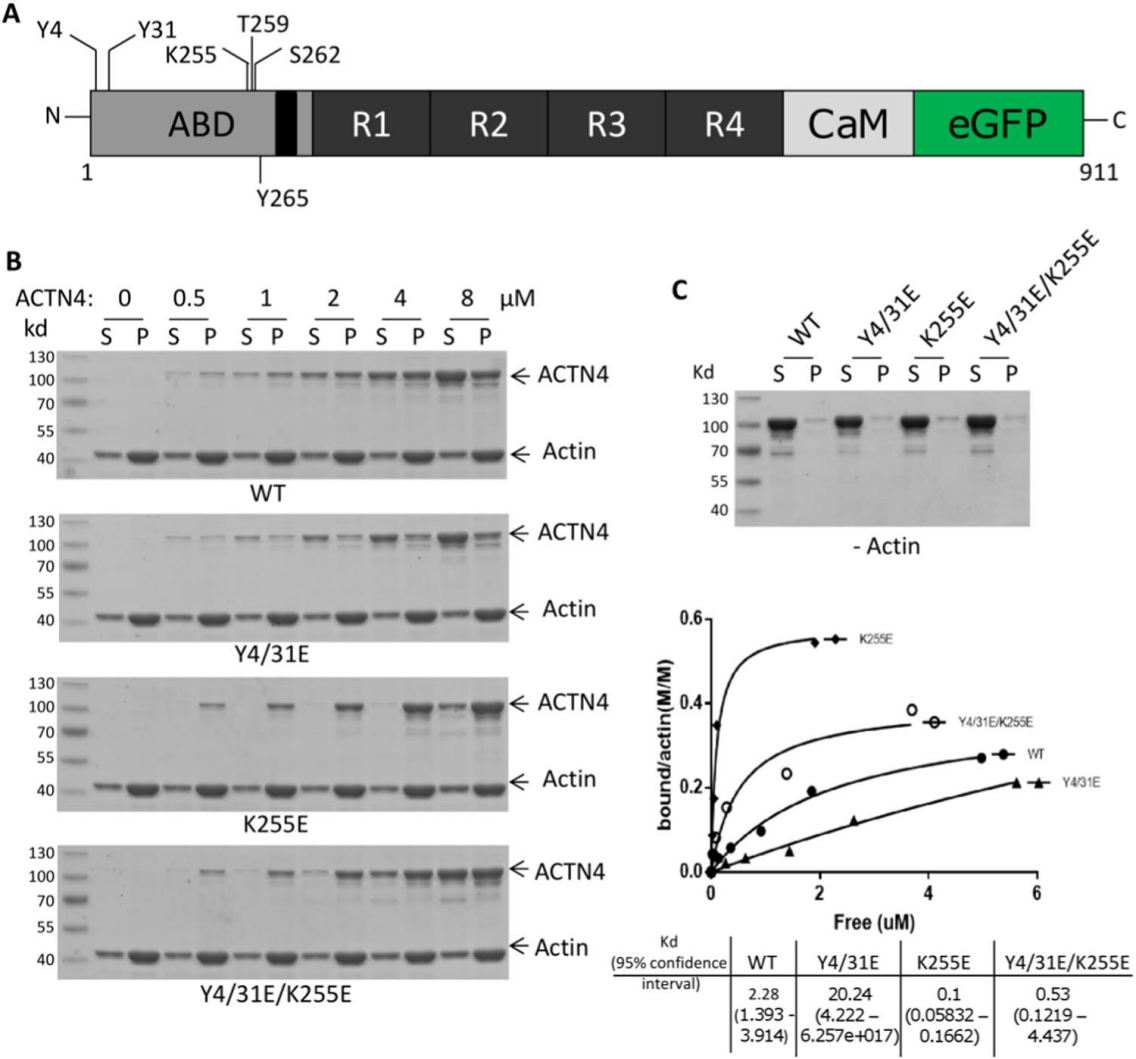
K255E ACTN4 actin binding is modulated by the amino terminus. (**A**) Schematic of human ACTN4 tagged at its carboxyl terminus. ABD: actin binding domain, R: spectrin repeat; CaM: calcium binding motif; eGFP: enhanced green fluorescent protein. All mutations are marked. (**B**) Full length of WT and mutant ACTN4 were expressed in and purified from *E.coli*. ACTN4 sedimentation assay was performed in the presence of F-actin. The image shown is of coomassie-stained SDS-PAGE. S stands for the supernatant and P stands for the pellet of centrifuged reaction. Saturation binding curves were generated by nonlinear regression from quantified results of Coomassie-blue-stained polyacrylamide gels. Calculation of *K*d, and 95% confidence intervals were calculated by using GraphPad Prism 7 software. (**C**) SDS gel of WT and mutant ACT4 protein from actin binding assay in the absence of actin. Shown experiments are representative of three independent experiments.

The pathological mutation of the lysine to a glutamic acid at position 255 of ACTN4 results in tight binding to actin filaments and leads to an autosomal dominant form of focal segmental glomerulosclerosis^13^. As this was the first, and remains the most studied FSGS mutation, we used this one as the test mutation. Compared to ACTN1, ACTN4 has unique and unstructured N-terminal tail amino acids which have been suggested to regulate its actin binding activity (Fig. 1A). We previously found that the actin binding cleft of wild type ACTN4 is latched when EGF stimulation leads to phosphorylation on tyrosines 4 and 31^6^ and this prevents F-actin access and thus binding. We thus attempted to determine if EGF-mediated phosphorylation of ACTN4 at tyrosines 4 and 31 affects the F-actin binding activity of K255E. Y4/31E/K255E, a triple mutation ACTN4 was constructed to mimic the putative EGF-induced tyrosyl phosphorylated K255E.

We then expressed and purified WT, Y4/31E, K255E and Y4/31E/K255E from *E. coli* and tested actin filament binding *in vitro*. In agreement with our postulate, the F-actin binding activity of Y4/31E/K255E was significantly reduced compared to K255E (Fig. 1B). The differential co-sedimentation with F-actin was not due to protein aggregates, as only trace amounts were sedimented in the absence of F-actin, and these were consistent between WT and the mutant ACTN4 (Fig. 1C). These trace levels were accounted when calculating affinities. While the F-actin binding affinity of the K255E ACTN4 was reduced by additional Y4/31E mutations, the restoration was only partial compared to WT ACTN4 (Fig. 1B).

**Figure 2:**
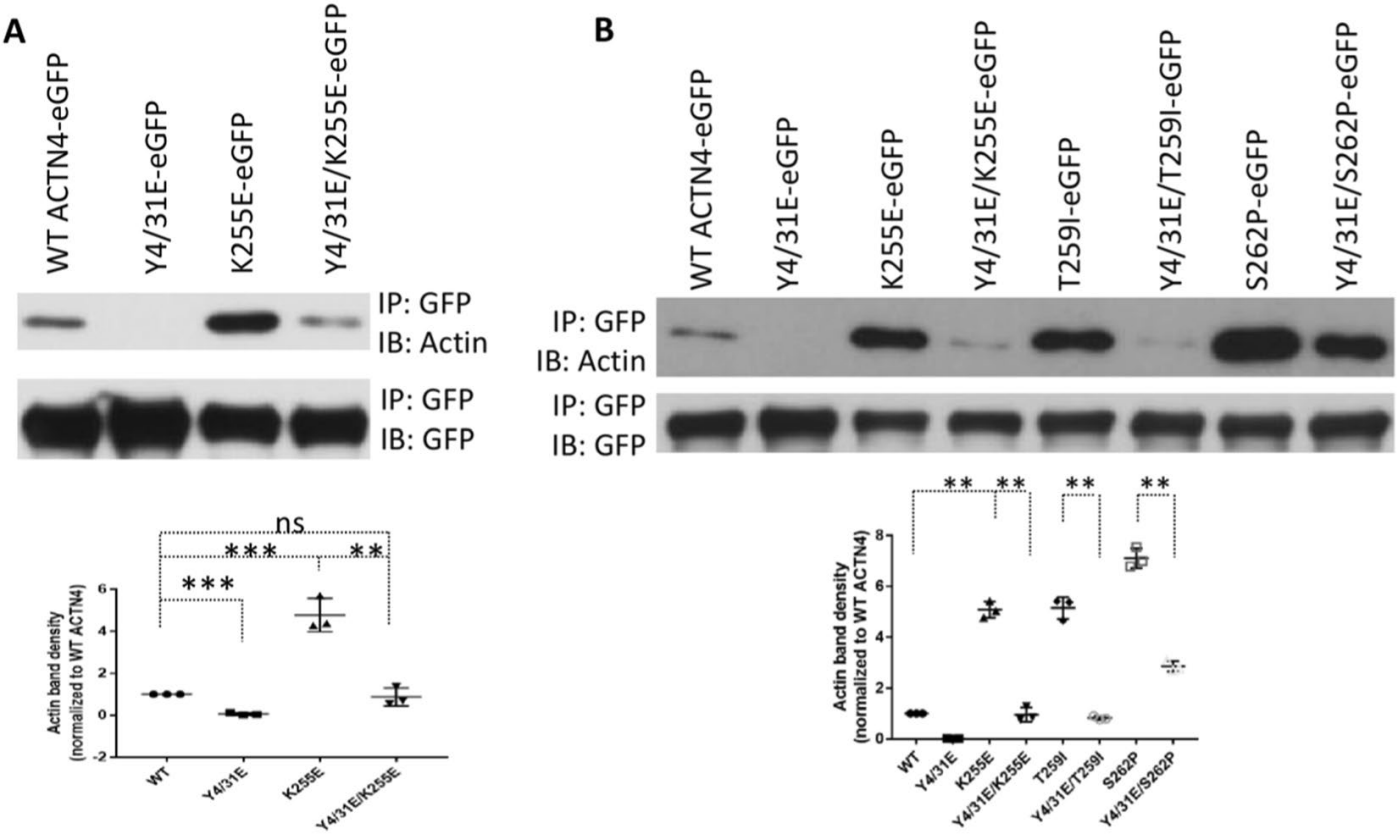
Phosphomimetic mutations restore the F-actin binding activities of FSGS-associated ACTN4. (**A,B**) Immunoblotting of co-immunoprecipitated F-actin and immunoprecipitated GFP-tagged WT and mutant ACTN4. Shown is representative of three independent experiments. The density of actin bands is shown in graph. Data are mean of ±SD of three independent experiments. Statistical analysis was performed using Student’s t-test. **p < 0.01, ***p < 0.001.

To exclude the possibility of different actin binding activity of ACTN4 mutants being caused by incorrect/mis-folding of proteins during expression and purification from *E. coli*, we transiently expressed WT and mutants tagged with eGFP at their carboxyl termini both to observe the localization in cells and also for co-immunoprecipitation of F-actin using GFP antibody. Consistent with actin binding *in vitro*, K255E ACTN4 co-precipitated with excessive amounts of F-actin compared to WT ACTN4 (Fig. 2A). Adding the Y4/31E phosphomimetic mutations to WT diminished the co-precipitation of F-actin to trace amounts if any. The triple mutation Y4/31E/K255E ACTN4 significantly reduced the amount of F-actin co-precipitated compared to K255E ACTN4.

It is possible that these findings are unique to the K255E ACTN4 found in the initial case. To ascertain the generalizability to other FSGS mutations, we looked at other FSGS families that provided for two more substitutive mutations, T259I and S262P that occurred within the actin binding domain of ACTN4. These mutants also present elevated actin binding activities^7^. To further confirm the effect of Y4/31E on the biological and cellular regulation of ACTN4 mutations in FSGS, we constructed Y4/31E/T259I and Y4/31E/S262P ACTN4 and determined their F-actin binding activities using co-immunoprecipitation. As shown in Fig. 2B, both the T259I and S262P ACTN4 co-precipitated F-actin similarly to K255E ACTN4. The addition of the phosphomimetic mutations Y4/31E reduced this association to different degrees. Y4/31E/T259I interacted with F-actin at a similar level with Y4/31E/K255E ACTN4 and WT ACTN4. Interestingly, the Y4/31E/S262P ACTN4 retained excessive actin association, though it was also significantly downregulated compared to S262P ACTN4. Taken together, these results suggest that phosphomimetic mutations of K255E, T259I and S262P at tyrosines 4 and 31 decrease their bindings to actin filaments.

### Phosphomimetic mutations of K255E, T259I and S262P ACTN4 on Y4/31 affect their distributions in cells

**Figure 3:**
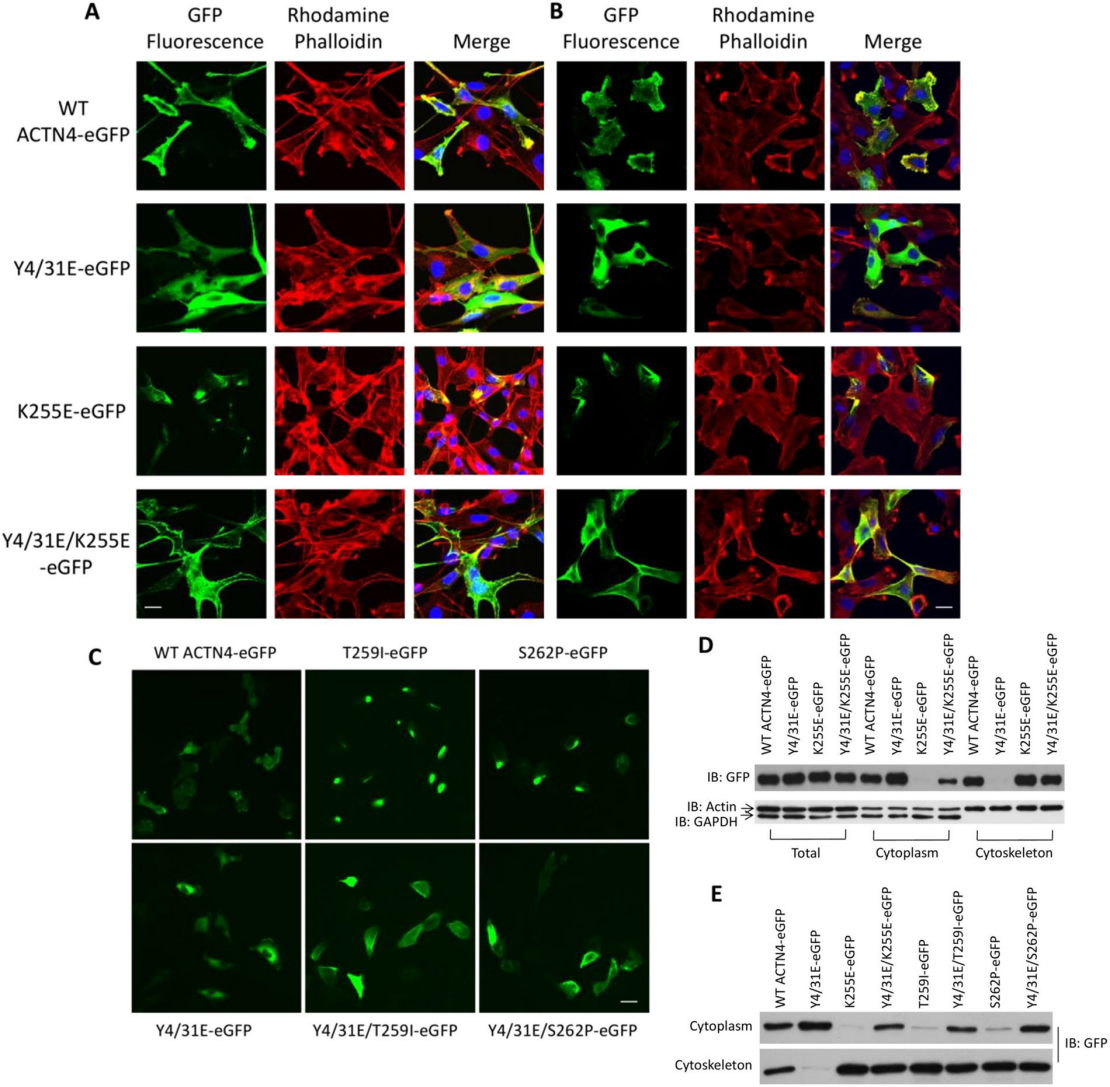
Cellular localizations of FSGS-associated ACTN4 are regulated by amino terminus phosphomimetics mutations. **(A–C)** Cells grown in 6-well plate were transfected with indicated plasmids and then stained with rhodamine phalloidin for actin filaments and DAPI for nuclei. Green is GFP-tagged ACTN4. (A) is NR6WT fibroblasts and (**B,C**) are melanoma cell line WM1158. *Scale bar*, 20 μm. **(D,E)** Immunoblotting of GFP, actin and GAPDH in total cellular lysate, fraction of cytoplasm and cytoskeleton. All WT and indicated mutants are tagged with GFP at their C-terminus. Shown is the representative of three independent experiments.

K255E aggregates in fibroblasts due to its enhanced actin binding activity^13^. Our above findings showed that phosphomimetic mutations of the three tested FSGS ACTN4 at tyrosine 4 and 31 decreased their F-actin binding activities. We thus determined if phosphomimetic mutations of K255E and other FSGS ACTN4 affect cellular localization. As shown in Fig. 3A, WT ACTN4 mostly localized at the cell edge and colocalized with actin filaments in the lamellipodia. In contrast but consistent with little to no actin binding, the Y4/31E ACTN4 was diffusely localized in the cytoplasm. In line with the previous report^13^, K255E ACTN4 forms bright fluorescent dots that were highly colocalized with actin filament. Notably, those bright fluorescent spots were no longer obvious in cells in which Y4/31E/K255E ACTN4 was expressed. Instead, Y4/31E/K255E ACTN4 tended to reside in cytoplasm and on the cell edge similar to WT ACTN4.

To determine if the cellular localizations are specific to a cell type, we transiently transfected WT and mutant ACTN4 into melanoma cell line WM1158, as ACTN4 is critical for melanoma invasion^20^. We found that the localization pattern of WT and all mutant ACTN4 were very similar to their localizations in fibroblasts (Fig. 3B).

Similar to K255E ACTN4, both T259I and S262P ACTN4 also presented small bright green fluorescent spots in WM1158 cell (Fig. 3C). In contrast, there were no obvious small bright spots in WM1158 cells in which Y4/31E/T259I and Y4/31E/S262P ACTN4 were expressed.

The above data were taken to implicate tight actin filament binding, with limited turnover, results in intracellular aggregations of the FSGS ACTN4 mutations. To further demonstrate that these cellular localizations were due to enhanced or reduced binding activity to the cytoskeleton, the cell was fractionated into cytoskeleton and cytosol. As shown in Fig. 3D, WT ACTN4 was found in both fractions with the Y4/31E ACTN4 residing almost exclusively in the cytoplasm. K255E ACTN4 presented the opposite pattern, being almost totally with the cytoskeleton. The inclusion of the phosphomimetic Y4/31E mutations into the K255E ACTN4 did liberate a fraction of the protein to reside in the cytoplasm. The other FSGS mutations T259I and S262P ACTN4 behaved similarly to K255E ACTN4, being bound essentially fully to cytoskeleton (Fig. 3E). Similar to Y4/31E/K255E ACTN4, Y4/31E/T259I and Y4/31E/S262P ACTN4 were distributed between the cytoskeleton and cytoplasm. Taken together, phosphomimetic mutations of K255E, T259I and S262P ACTN4 at tyrosine 4 and 31 significantly reduced the aggregates with F-actin filaments in cells via looser association to the cytoskeleton.

### Phosphomimetic mutations of K255E, T259I and S262P ACTN4 on Y4/31 restore their functions on cell motility

**Figure 4:**
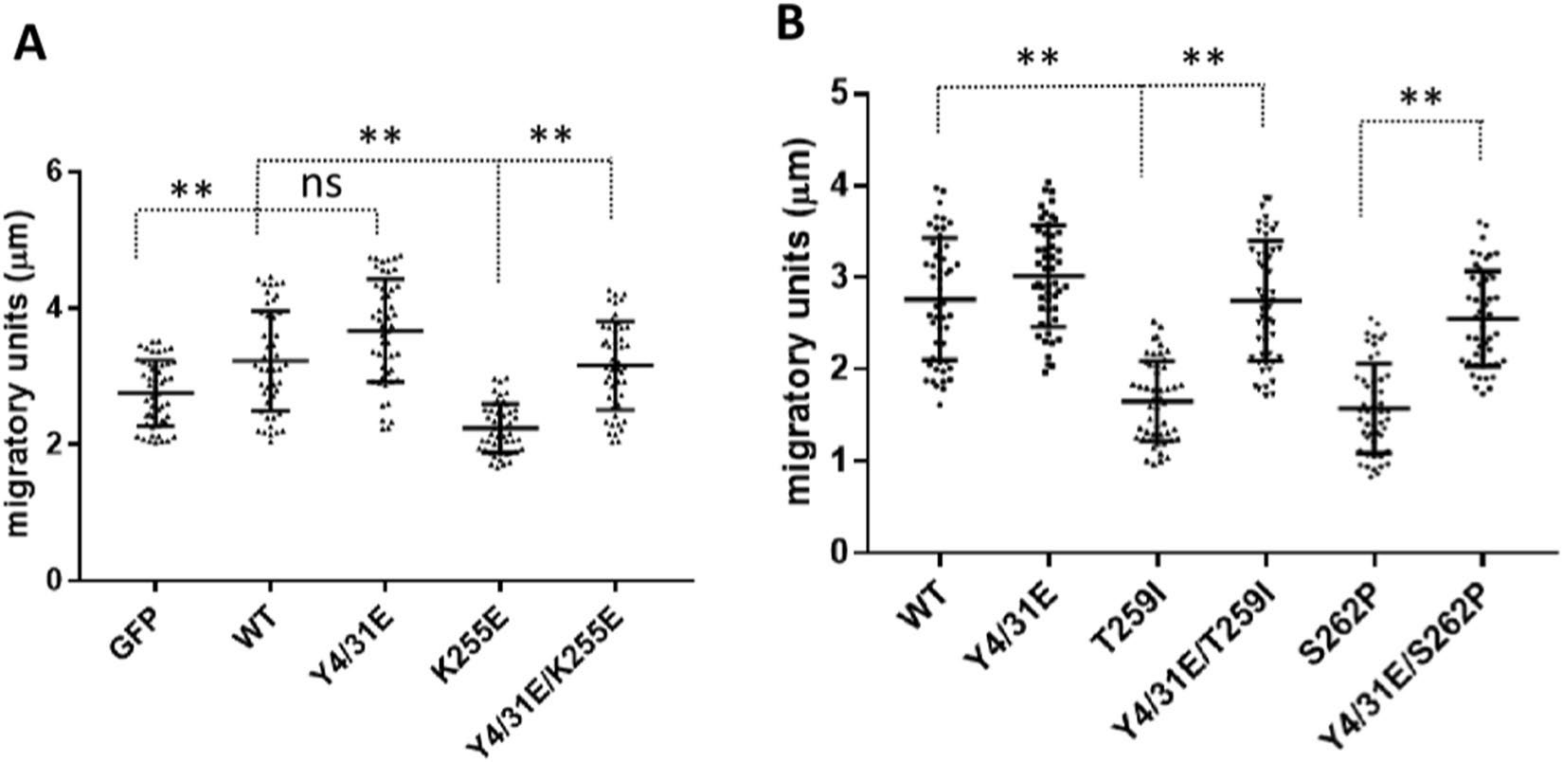
Cell migration speeds of FSGS-associated ACTN4 are increased by amino terminus phosphomimetic mutations. **(A,B)** Melanoma cells WM1158 were transiently transfected with indicated plasmids tagged with GFP for 16 h with the GFP positive cells being tracked for 6 h with an interval of 10 min. The migration speed of each individual cell was calculated using MetaMorph software. The migratory units stand for the average distance of cells moved within 10 minutes (this was chosen as the interval of measurement). Cell number (N) = 50. Data are mean of ±SD of three independent experiments. Statistical analysis was performed using Student’s t-test. ns: no statistical difference, **p < 0.01.

We next examined functional consequences of the altered actin filament interactions. Ehrlicher *et al*. had reported that K255E ACTN4 inhibited cell migration^21^. Thus, we performed a live cell tracking assay to determine if phosphomimetic mutation of K255E increases cell migration. As shown in Fig. 4A, overexpression of WT ACTN4 significantly enhanced the cell migration speed of WM1158 compared to GFP itself which is in line with previous findings^20^. Overexpression of K255E ACTN4 in WM1158 cells resulted in a significant decrease in cell migration speed compared to either GFP itself or WT ACTN4. Notably, cells overexpressing Y4/31E/K255E ACTN4 migrated at a similar speed with WT ACTN4, and significantly faster than K255E ACTN4. Similarly, both T259I and S262P ACTN4 decreased cell motility, with the inclusion of the phosphomimetic mutation Y4/31E rescuing this inhibitory phenotype (Fig. 4B). These findings further support the more physiological functioning of the K255E, T259I and S262P ACTN4 upon putative phosphorylation by growth factors, partially reverting their strong affinities for F-actin to enable cytoskeletal turnover and thus enhanced cell motility, as suggested by the more physiological localization (Fig. 3).

### Phosphomimetic mutation of Y265E ACTN4 on Y4/31 partially affects its F-actin binding activity, cytoplasmic distribution and cell motility

**Figure 5:**
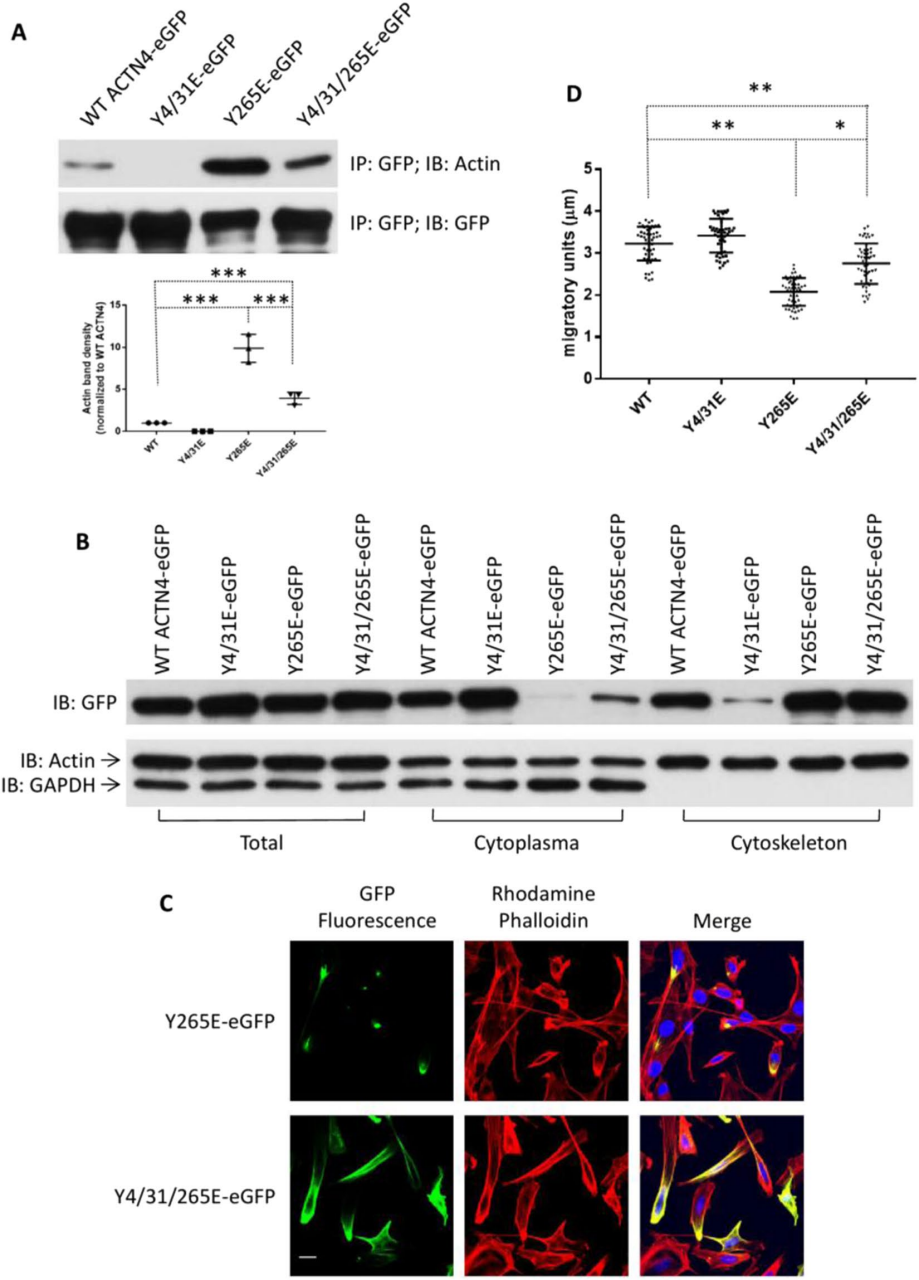
Actin binding activity of Y265E ACTN4 is partially regulated by amino terminus phosphomimetic mutations. **(A)** Immunoblotting of immunoprecipitated WT ACTN4-eGFP and ACTN4Y265E-eGFP using indicated antibodies. **(B)** Immunoblotting of GFP, actin and GAPDH in total cellular lysate, fraction of cytoplasm and cytoskeleton. **(C)** Staining of NR6WT cells transiently transfected with Y265E-eGFP or Y4/31/265E-eGFP. Red:  Rhodamine phalloidin; Green: fluorescence of ACTN4-eGFP; Blue: DAPI. *Scale bar*, 20 μm. **(D)** Cell migration speed of melanoma WM1158 cells transiently expressing WT or indicated eGFP-tagged mutants tracked using live microscopy and analyzed using MetaMorph software. The migratory units stand for the average distance of cells moved within 10 minutes. Cell number (N) = 50. Images are a representative of three independent experiments. Data are mean of ±SD of three independent experiments. Statistical analysis was performed using Student’s t-test. ***p < 0.001.

Above findings suggest that phosphomimetic mutations of ACTN4 at tyrosine 4 and 31 downregulate the bindings of K255E, T259I and S262P ACTN4 to actin filaments and allow a more physiological behavior of the cells containing these molecules. To further confirm this effect, another artificial ACTN4 mutant Y265E attracted us based on the following reasons: (i) We previously noted that Y265E, a phosphomimetic ACTN4 construct, bound actin filaments very tightly resulting in the formation of Y265E ACTN4 clusters in fibroblasts^15^ (Fig. 5C); (ii) Y265E ACTN4 binds to actin filaments more avidly than K255E ACTN4; (iii) Y265 also resides within F-actin binding domain and has been proposed as a phosphorylation target site^22,23^. Therefore, we also tested if phosphomimetic mutation of Y265E ACTN4 at tyrosine 4 and 31 also decreases its actin binding activity and limits the formation of aggregates with actin filaments in cells. As shown in Fig. 5A, the content of actin co-precipitated with Y4/31/265E ACTN4 is significantly decreased compared to Y265E ACTN4. In line with co-precipitation findings, Fig. 5B revealed that almost all Y265E ACTN4 presented in the cytoskeleton fraction and Y4/31/265E ACTN4 partially partitioned to the cytoplasmic fraction as reflected by cellular localization (Fig. 5C). Next, we performed live cell tracking assay to determine the effects of Y265E and Y4/31/265E ACTN4 expression on cell motility of melanoma cells. We found that overexpression of Y265E ACTN4 dramatically inhibited cell migratory speed but a phosphomimetic mutation at tyrosine 4 and 31 significantly limited the inhibition of Y265E on cell migration (Fig. 5D). These findings suggest that phosphomimetic mutation of Y265E in the unstructured N-terminus also efficiently closes the F-actin binding domain of Y265E ACTN4 resulting in a decrease in the binding to actin filaments and restoring more physiological behavior.

### Phosphomimetic mutations of K255E, T259I and S262P ACTN4 on Y4/31 affect their cellular distribution and migration in podocytes

**Figure 6:**
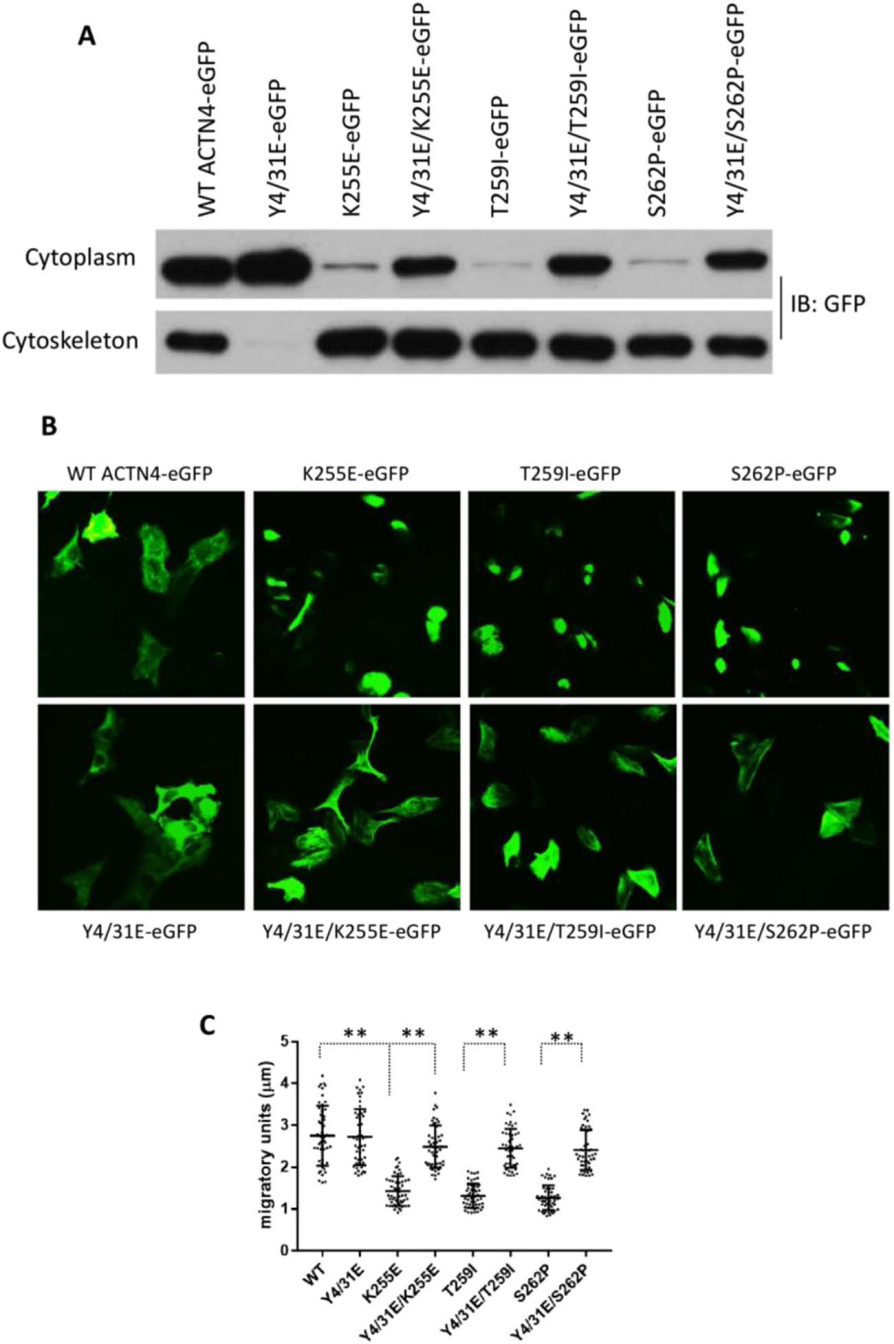
Phosphomimetic mutations regulate the function of FSGS-associated ACTN4 in podocytes. **(A)** All WT and indicated ACTN4 mutants are tagged with GFP at their C-terminus and transiently expressed in podocytes. Immunoblotting of GFP-tagged ACTN4 in the fraction of cytoplasm and cytoskeleton. Shown is  representative of three independent experiments. **(B)** Localization of GFP tagged WT and mutants in podocytes. *Scale bar*, 20 μm. **(C)** Migratory graph of podocytes transiently expressing either WT or indicated mutants. The migration speed of each individual cell was calculated using MetaMorph software. The migratory units stand for the average distance of cells moved within 10 minutes. Cell number (N) = 50. Data are mean of ±SD of three independent experiments. Statistical analysis was performed using Student’s t-test. **p < 0.01.

FSGS related mutations affect the function of kidney due to their aggregations in podocytes in which ACTN4 is the overwhelmingly predominant alpha-actinin. Thus, whether the effects of these mutations on podocytes functioning can be regulated by amino-terminal modifications is critical to understanding how the podocytes can function in FSGS. As shown in Fig. 6A, the FSGS mutant ACTN4 all presented tight cytoskeleton binding with negligible amounts in the cytoplasm. Introduction of the phosphomimetic mutations Y4/31E increased the cytoplasmic fraction of all Y4/31E/K255E, Y4/31E/T259I and Y4/31E/S262P ACTN4. Again as in fibroblasts and melanoma cells, the K255E, T259I, and S262P ACTN4 all formed clusters in podocytes, whereas the Y4/31E/K255E, Y4/31E/T259I, and Y4/31E/S262P ACTN4 were no longer aggregated (Fig. 6B). Most importantly, we found that podocytes transiently overexpressing Y4/31E/K255E, Y4/31E/T259I or Y4/31E/S262P ACTN4 migrated much faster than their non-phosphomimetic mutant ACTN4 (Fig. 6C). These results suggest that FSGS related mutations of ACTN4 within its F-actin binding domain affect cellular localization and motility of podocytes.

### EGF-mediated phosphorylation of K255E ACTN4 restores its localization in fibroblasts

Our above findings were based on introducing a phosphomimetic mutation, Y4/31E, into the ACTN4 variants. This was necessary as tyrosyl phosphorylation upon exposure of the cells to EGF and other growth factors results in substoichiometric phosphorylation of only a small fraction of the ACTN4 molecules^6,15^. For the *in vitro* experiments the replacement of tyrosine with glutamic acid (E), routinely used to mimic the phosphorylation of proteins at tyrosines, was necessary; even *in vivo* the expression of these mutations allowed for dominant behavior. However, this substitution does not occur in mutant ACTN4 and it overestimates the fractional phosphorylation^15^. Therefore, we asked if EGF-induced phosphorylation at tyrosine 4 and 31 of the FSGS ACTN4 may occur. K255E ACTN4 was chosen as the index mutant. Prior modeling suggests that the intrinsically disordered region is accessible only if ACTN4 is not bound to actin^6^. Thus, the question arises of whether physiologically-induced phosphorylation would overcome the tight binding of K255E ACTN4 to actin filaments and restore some functionality. Relocalization to the cytoplasm and membrane would reflect this.

**Figure 7:**
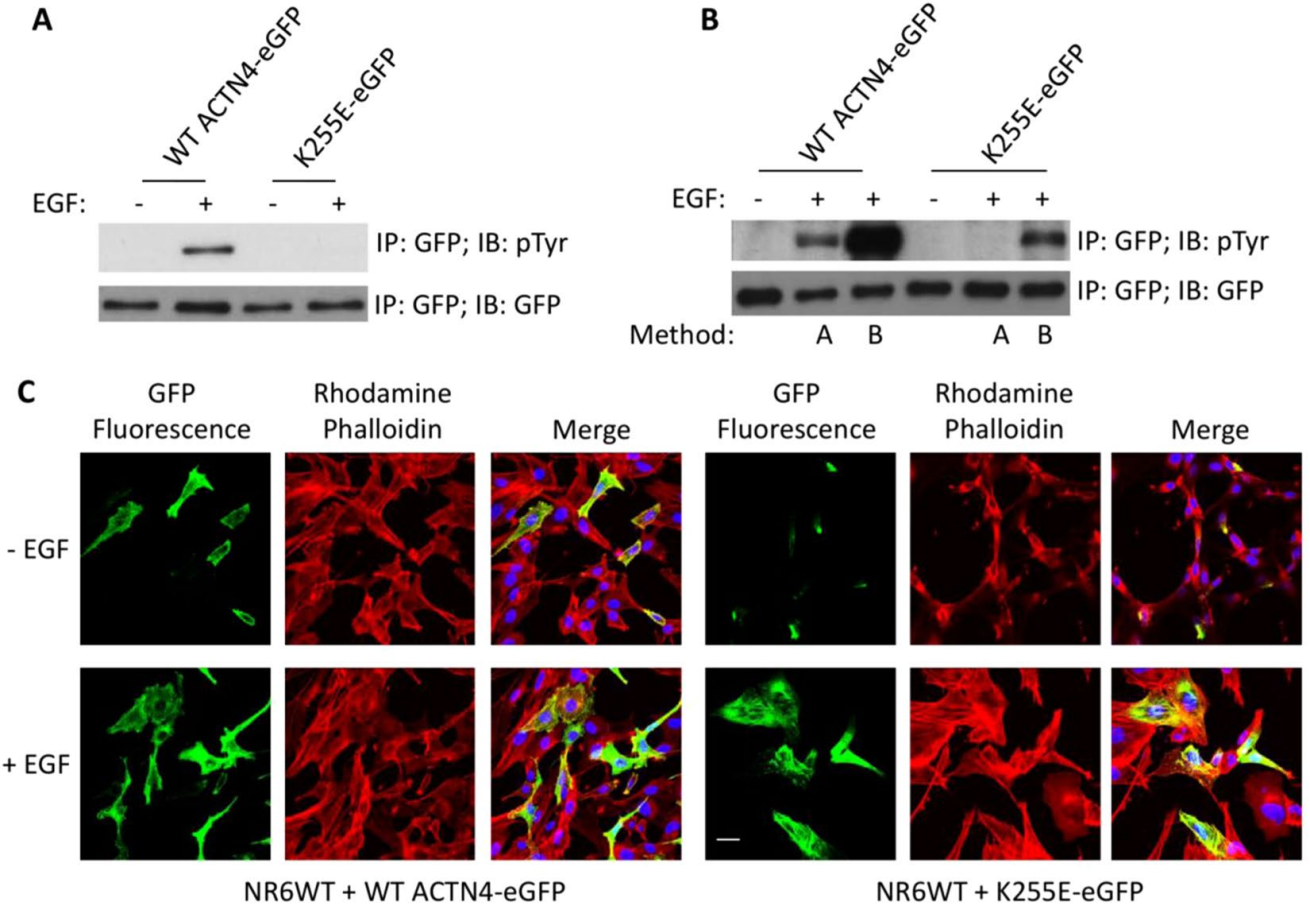
Continuous EGF stimulation triggers the phosphorylation of K255E ACTN4 and alters its cellular localization. **(A)** Immunoblotting of immunoprecipitated WT ACTN4-eGFP and K255E-eGFP from NR6WT fibroblasts pretreated with 100 μM sodium vanadate for 30 min followed by 10 nM EGF stimulation for 10 min. **(B)** Immunoblotting of immunoprecipitated WT ACTN4-eGFP and K255E-eGFP from NR6WT fibroblasts using indicated antibodies. Method A stands for cells transiently transfected for 16 h were pretreated with 100 μM sodium vanadate for 30 min followed by 2 nM EGF stimulation for 10 min prior to lysing for immunoprecipitation. Method B stands for cells transfected for 2 h were pretreated with 100 μM sodium vanadate for 30 min followed by stimulation with 2 nM EGF per every hour for a duration of 6 h prior to lysing for immunoprecipitation. Images in (**A,B**) are representative of three independent experiments. **(C)** Staining of NR6WT cells transfected with K255E-eGFP and treated with 2 nM EGF using method B. Red: Rhodamine phalloidin; Green: fluorescence of K255E-eGFP; Blue: DAPI. *Scale bar*, 20 μm.

To answer this question, we stimulated fibroblasts in which K255E ACTN4 was transiently transfected and expressed for 16 h, with 2 nM EGF added for the final 10 min (method A: transfection for 16 h and then 2 nM EGF for additional 10 min) prior to harvesting for immunoprecipitation against GFP antibody followed by immunoblotting against phosphotyrosine to detect the phosphorylation. As shown in Fig. 7A, there was negligible or no detectable phosphorylation on K255E ACTN4, while the control WT ACTN4 was vigorous phosphorylated. This suggested that actin filament bound K255E ACTN4 was not accessible for phosphorylation as we previously noticed that replacement of tyrosine 265 with glutamic acid quenched the phosphorylation of ACTN4 at tyrosine 4 and 31 mediated by EGF stimulation^15^ concurrent with a similarly large increase in its actin binding activity.

We postulate that the reason why EGF stimulation did not lead a phosphorylation of K255E ACTN4 was that it bound actin filament too tightly; this was shown by increased EGF-mediated phosphorylation of Y265E ACTN4 that were on release from actin filaments prior to stimulating by EGF using Latrunculin A^15^. Therefore, we stimulated cells starting at 2 h after transfection with K255E ACTN4 with 2 nM EGF as described in method B (method B: transfection for 2 h and then 2 nM EGF stimulation every hour for a duration of 6 h). Surprisingly, we observed a significant phosphorylation of K255E ACTN4 in method B compared to method A (lane 6 vs lane 5) suggesting that there can be a physiological phosphorylation of K255E ACTN4 at tyrosine 4 and 31 in cells stimulated with EGF (Fig. 7B). Next, we treated K255E ACTN4-expressing cells using method B and found that EGF stimulation caused a dramatic change with a cytoplasmic distribution of fluorescent K255E ACTN4 compared to aggregation in both no EGF treatment in method B and EGF treatment in method A (Fig. 7C). These results suggest that EGF-mediated phosphorylation of K255E ACTN4 decreases its binding to actin filaments.

## Discussion

ACTN4 has been originally identified as an actin crosslinking protein^24^. Besides crosslinking actin filaments, its biological function has been expanded to be involved in bridging actin filaments/cytoskeleton to the plasma membrane, taking part in gene transcription, and interacting with other proteins^25–28^. However, the regulation of the actin binding activity of ACTN4 in cells is still primary as the majority of ACTN4 exists in an anti-parallel homodimer which presents actin binding domains at both ends for crosslinking actin filaments or bridging to the membrane^29^. Naturally-occurring mutations such as K255E, T259I and S262P ACTN4 cause focal segmental glomerulosclerosis (FSGS) in affected humans because of their elevated actin binding activities^13^. Our present findings for the first time reveal that the regulatory phosphorylations of K255E, T259I and S262P ACTN4 at tyrosine 4 and 31 significantly loosen the F-actin binding back towards wild type levels, suggesting that the N-terminus of ACTN4 plays critical role in the regulation of the F-actin binding activity.

Besides the function of ACTN4 on the regulation of cytoskeleton, it is also involved in the formation of focal adhesion^20^. Immunofluorescence of paxillin shows that there is no obvious large focal adhesion bars within K255E ACTN4 aggregates while WT ACTN4 predominantly colocalizes with paxillin in podocytes (Fig. S1A). Therefore, the effect of K225E and other FSGS mutant ACTN4 on the inhibition of cell motility may be largely due to their tight binding in aggregates to actin filaments (Fig. S1B). Indeed, we observed that K255E and Y265E ACTN4 were always aggregated during cell motility while Y4/31E/K255E and Y4/31/265E ACTN4 were dynamically distributed within the cells, even though Y4/31/265E ACTN4 aggregation was only partial disrupted (Fig. S2A). This phenomenon can also help to explain why Y4/31/265E ACTN4 overexpressing cells migrated slower than those Y4/31E/K255E ACTN4 overexpressing cells (Fig. S2A,B).

To exclude the possibility of actin binding activity and cellular distribution of the FSGS mutants was not cell type dependent, fibroblasts and melanoma cells were tested. The mutant ACTN4 presented similar actin binding activity and cellular distribution in both cell types, and in podocytes. The inhibitory effects on cell motility and the rescue by the phosphomimetic mutation Y4/31E also presented similarly in both cells demonstrating a molecule-intrinsic effect. Most importantly, we found that the effect of the phosphomimetic mutations tyrosine 4 and 31 in all K255E, T259I, and S262P ACTN had the similar effects on the regulation of F-actin binding activity and cell motility in podocytes as in fibroblasts and melanoma.

EGF stimulation triggers the phosphorylation of ACTN4 at tyrosine 4 and 31^15,19^. However, we found that ACTN4 can be rapidly phosphorylated once cells are stimulated for seconds. On the other hand, K255E and Y265E ACTN4 that are bound to actin filaments were not capable of being phosphorylated via EGF stimulation implying that the phosphorylation sites tyrosine 4 and 31 becomes unavailable for protein kinases due to their conformational change during the interaction with actin filaments. Indeed, compared to the significant phosphorylation of WT ACTN4, the phosphorylation of both K255E and Y265E ACTN4 is almost non-detectable (Fig. S3A, method A lanes in top panel,) but continuous EGF stimulation of cells expressing nascent K255E or Y265E ACTN4 significantly increases phosphorylation at tyrosine 4 and 31 although the phosphorylation content of Y265E is still lower than K255E ACTN4 (Fig. S3A, method B lanes, and lane 9 vs lane 6); this concurs with an accumulation of the small fraction of unbound molecules (Fig. S3B). Additional supporting evidence comes from our previous finding that Y265E ACTN4 became phosphorylated once the cells were treated with Latrunculin A to disrupt actin filaments, releasing the bound ACTN4^15^. This would also cover the possibility that ACTN4 bound on cytoskeleton is too distal from cytoplasmic membrane where the necessary protein kinases needed for the phosphorylation of ACTN4 reside on to be phosphorylated.

Recently, Yee *et al*. claimed that the enhanced F-actin binding activity of K255E ACTN4 was due to its misfolding in endoplasmic reticulum (ER) during protein synthesis^30^. Treatment of K255E ACTN4 expressing cells with sodium 4-phenylbutyrate (4-PBA), a small chemical chaperone that has been suggested to improve protein folding by stabilizing the intramolecular hydrophobic bonds or lowering a protein’s free energy state significantly inhibited the formation of K255E ACTN4 clusters^30,31^. This raises another question whether all synthesized K255E ACTN4 are folded correctly during the drug treatment and if not, the misfolded K255E ACTN4 will still irreversibly bind to actin filaments to interrupt cell function. On the other hand, analysis of the crystal structure of ABD in the absence of actin reveals that K255E ACTN4 has same conformation as WT ACTN4^32^. Weins *et al*. also hypothesized that there is a conformational change when K255E ACTN4 interacts with actin filaments resulting in enhanced actin binding activity^13^, although high-resolution analysis of the crystal structure of the complex of full length WT/K255E ACTN4 bound to actin filaments is needed to better explain why K255E ACTN4 presents elevated actin binding activity.

The relationship between high avidity binding of ACTN4 mutants to actin filaments and the FSGS phenotype appears binary at present. The limited data granularity of disease symptoms and progression, and the similar coarseness of the measurements for actin filament binding and effects on cell migration, do not allow for a graduated correlation between mutant ACTN4 biochemical functions and human pathology. The number of definitive disease-causing ACTN4 mutations is small, and within families sharing the same mutations, variability in severity of disease is very large, making mutation-phenotype correlations difficult. This could be approached, in studies beyond the scope of the current missive, by knock-ins that combine the FSGS mutations^33^ with the phosphomimetic Y4/31E mutations; we would predict milder phenotypes. Obviously, this is not approachable in the persons afflicted, so that an intervention would be needed to loosen the actin filament binding of extant FSGS ACTN4 mutants. We propose that increasing the phosphorylation levels of the amino-terminal Y4 and Y31 should ameliorate the more severe disease aspects. Thus, agents that augment EGF signaling could be viewed as potential therapeutics for treating FSGS patients.

## Methods

### Reagents and cells

Murine fibroblasts (NR6WT) expressing human epidermal growth factor receptor and modified as previously described^4^, were grown in alpha-MEM media supplemented with 1x non-essential amino acids, 1x sodium pyruvate, 1x L-glutamine, 1x pen/strep antibiotics, and 7.5% fetal bovine serum. Parental Melanoma cell line WM1158 and ACTN4 knockdown WM1158 (WM1158 ACTN4 KD)^20^ cells were grown in DMEM (1 gL^−1^ glucose):L15 3:1 medium with 10% fetal bovine serum and 1x pen/strip antibiotics. Transfection reagent xFect was purchased from Clontech Life Technologies (Grand Island, NY). Monoclonal antibody against phosphorylated tyrosine (P-Tyr-100) and polyclonal GAPDH antibody were purchased from Cell Signaling Technology (Beverly, MA). Polyclonal GFP antibody was purchased from Abcam (Cambridge MA). Purified bovine muscle actin and polyclonal Actin antibody were purchased from Sigma Aldrich (St. Louis, MO).

### Mutagenesis and construction of expression vectors

Mutagenesis of ACTN4 to create Y4/31E and the various FSGS mutations, and construction of mammalian expression vector pEGFP-N1 and bacterial expression vector pET-28a were performed as previously described^15^. Mutations were confirmed by DNA sequencing.

### F-actin filament sedimentation assays

F-actin filament sedimentation assays of WT or ACTN4 mutants were performed using a previously described protocol^19^. In brief, purified ACTN4 protein (at concentrations indicated in the figures) was mixed with 20 µg of actin monomer (catalogue no. 3653; Sigma) in reaction buffer in a final volume of 40 µl for 1 h at room temperature; these conditions resulted in actin polymerization with F-actin generation. That over 80% of the actin generated filaments was demonstrated by this amount being found in the pelleted fraction in the figures. After centrifugation at 100,000 g for 40 min at 25 °C, supernatants (S) were carefully transferred to a new Eppendorf tube containing 10 µl 5x sample buffer. The pellets (P) were rinsed briefly and carefully with 1 ml ddH_2_O, and then completely dissolved in 50 µl of 1x SDS-sample buffer. Twenty microliters of supernatant and pellet extraction were loaded on an 8% SDS-PAGE. Protein bands were visualized by Coomassie Blue G-250 staining. After complete destaining, band intensities were quantified using ImageJ software. The net amount of ACTN4 protein bound to F-actin was calculated by subtracting the self-aggregated proteins. (The fraction of ACTN4 proteins that may have aggregated was determined by centrifugation in the absence of F-actin performed at a concentration of 2 µM.) Nonlinear regression analysis for one-site binding (hyperbola), calculation of *K*d, and 95% confidence intervals were calculated by using GraphPad Prism 7 software.

### Immunoprecipitation and co-immunoprecipitation

Immunoprecipitation of eGFP-tagged ACTN4 was performed as previously described^19^. In brief, cells expressing wild-type or mutant ACTN4-eGFP were treated with appropriate reagents for indicated time followed by protein extraction in RIPA buffer (50 mM Tris-HCl pH 8, 150 mM NaCl, 1% NP-40, 0.1% SDS, 0.5% sodium deoxycholate, 1 mM EDTA). Notably, when EGF was used, cells were pretreated with 300 µM sodium vanadate for 30 min followed by stimulation with 10 nM EGF for 10 min or longer for Method B (Fig. 7). After centrifugation, clear lysates were incubated with agarose beads conjugated with antibodies against GFP (GFP-Trap, ChromoTek, Planegg-Martinsried, Germany) for at least 8 h at 4 °C. Then beads were carefully washed with RIPA buffer for 3 times. For co-immunoprecipitation, the RIPA buffer was replaced with a co-immunoprecipitation buffer consisting of 20 mM Tris-HCL pH 7.4, 120 mM NaCl, 1% Triton X-100 and 1x protease inhibitor cocktails set V (Billerica, MA). Agarose beads were collected and completely washed with appropriate buffer were incubated with 2x SDS protein sample buffer to eluate bound proteins and boiled for 3 min. The soluble denatured proteins were then subjected to electrophoresis and immunoblotting.

### Immunoblotting

Immunoblotting was performed using a previously described protocol^19^. Briefly, cells cultured in 6-well tissue culture plates were transfected or treated with appropriate reagents for indicated times and then were washed briefly with PBS in the absence of calcium and magnesium. Then cells were lysed in RIPA buffer in the presence of 1x protease inhibitors cocktails set V and scraped for collection into microcentrifugation tube for brief sonication. After centrifugation at 13,000 g for 30 min at 4 °C, the supernatant was carefully transferred to a new microcentrifugation tube for determining the concentration of total proteins using Thermo Scientific Pierce BCA Protein Assay (Rockland, IL). Ten micrograms of total proteins were applied for SDS-PAGE electrophoresis followed by electro-transfer from gel to polyvinylidene difluoride (PVDF) membrane and immunoblotting with appropriate primary antibodies and second antibodies labelled with HRP according to standard immunoblotting protocols.

### Isolation of cytoskeleton

The isolation of the cytoskeleton and cytoplasm fractions was performed as previously described with a modification^34^. In brief, cells grown and transfected in 6-well plates were treated with trypsin and harvested in an Eppendorf tube. Then cells were completely resuspended in 200 µl of lysis buffer consisting of 100 mM PIPES pH6.8, 1 mM MgCl2, 20 mM benzamine, 2.5 mM EGTA, 0.5% Triton X-100. After sitting at room temperature for 2 min, lysates were spun at 15000 g for 5 min to precipitate the cytoskeleton fraction. The supernatant (cytoplasm and membrane) was carefully collected and the pellet was then completely resuspended in 1 ml washing buffer (lysis buffer without 0.5% Triton X-100) for two times washing. To completely dissolve the pellet, 2 µl of 8 M urea was added to the pellet prior to addition of 200 µl of lysis buffer. Finally, one quarter volume of 5x SDS sample buffer was added to fractions of supernatant and cytoskeleton, respectively for SDS gel electrophoresis and immunoblottings.

### Transfection

Transfection and selection of stable cell lines were performed using a previously described protocol^19^. Briefly, approximately 90% confluent cells were incubated with a mix of DNA plasmid and xFect polymer in the quiescence (0.1% FBS for NR6WT cells) or low serum (2.5% FBS for WM1158 cells) media at 37 °C in a humidified incubator with 5% CO_2_ overnight.

### Cell tracking assay

Live cell tracking assay was performed as previously described^18^. Briefly, cells were transiently transfected with WT or mutant ACNT4-eGFP plasmid for 16 h. Next day, fluorescent images of transfected cells incubated in a 37 °C chamber with 5% CO_2_ were taken every 10 min for duration of 6 h under Nikon live cell fluorescent microscopy. Six to ten different positions were chosen for imaging dependent on the density of positive fluorescent cells. The velocity of all green cells was determined using the application of “track object” of MetaMorph software.

## Supplementary information

Supplementary information

Supplementary information

Supplementary information

Supplementary information

Supplementary information

Supplementary information
