# Supplementary material for: Focal segmental glomerulosclerosis ACTN4 mutants binding to actin: regulation by phosphomimetic mutations
Source: Sci Rep. 2019 Oct 29;9:15517. doi: 10.1038/s41598-019-51825-2 (PMC6820738; doi:10.1038/s41598-019-51825-2)
Supplement: Supplementary file 1 — Supplementary information [file 41598_2019_51825_MOESM1_ESM.pdf]

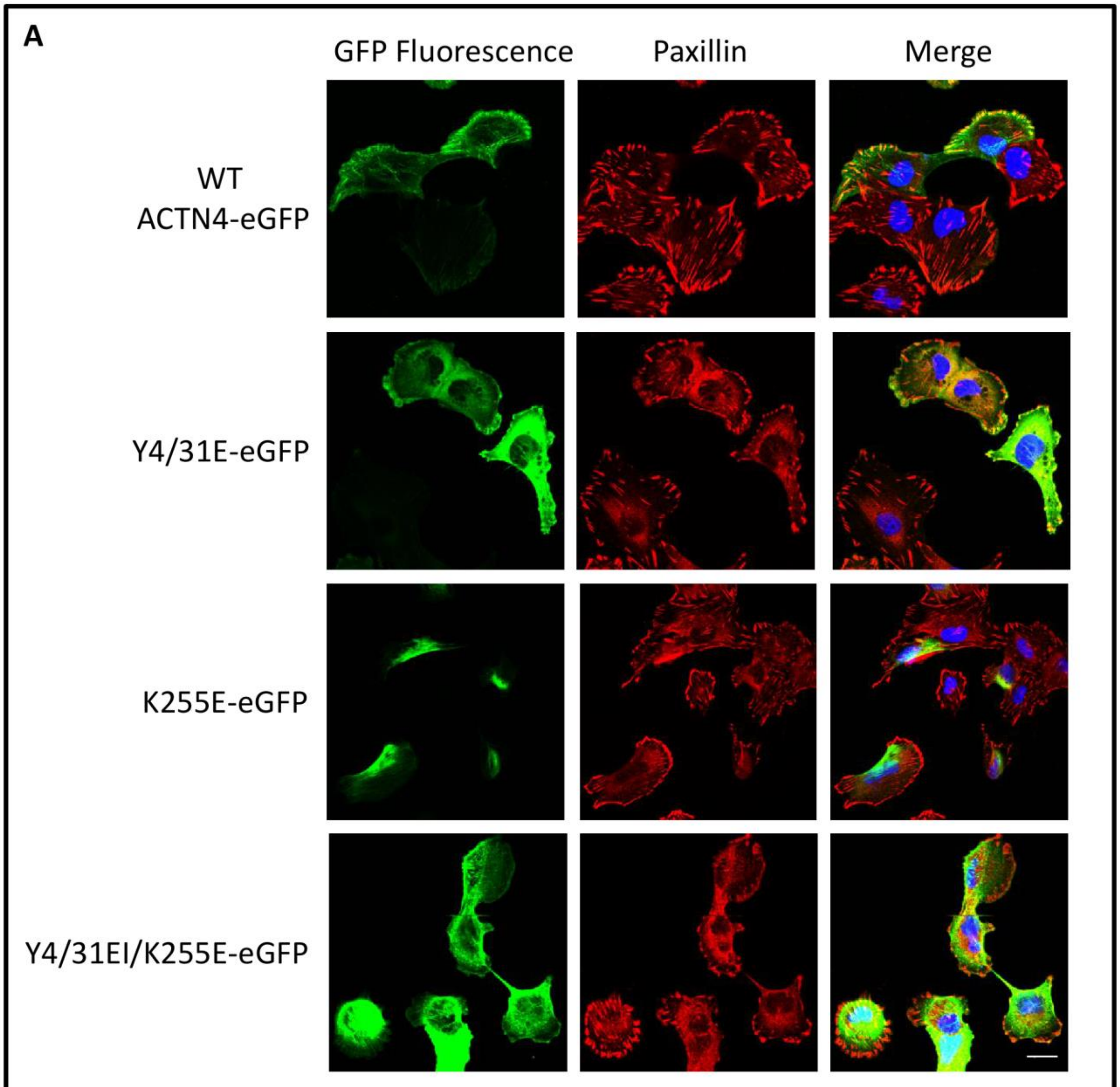

Supplementary Figure 1

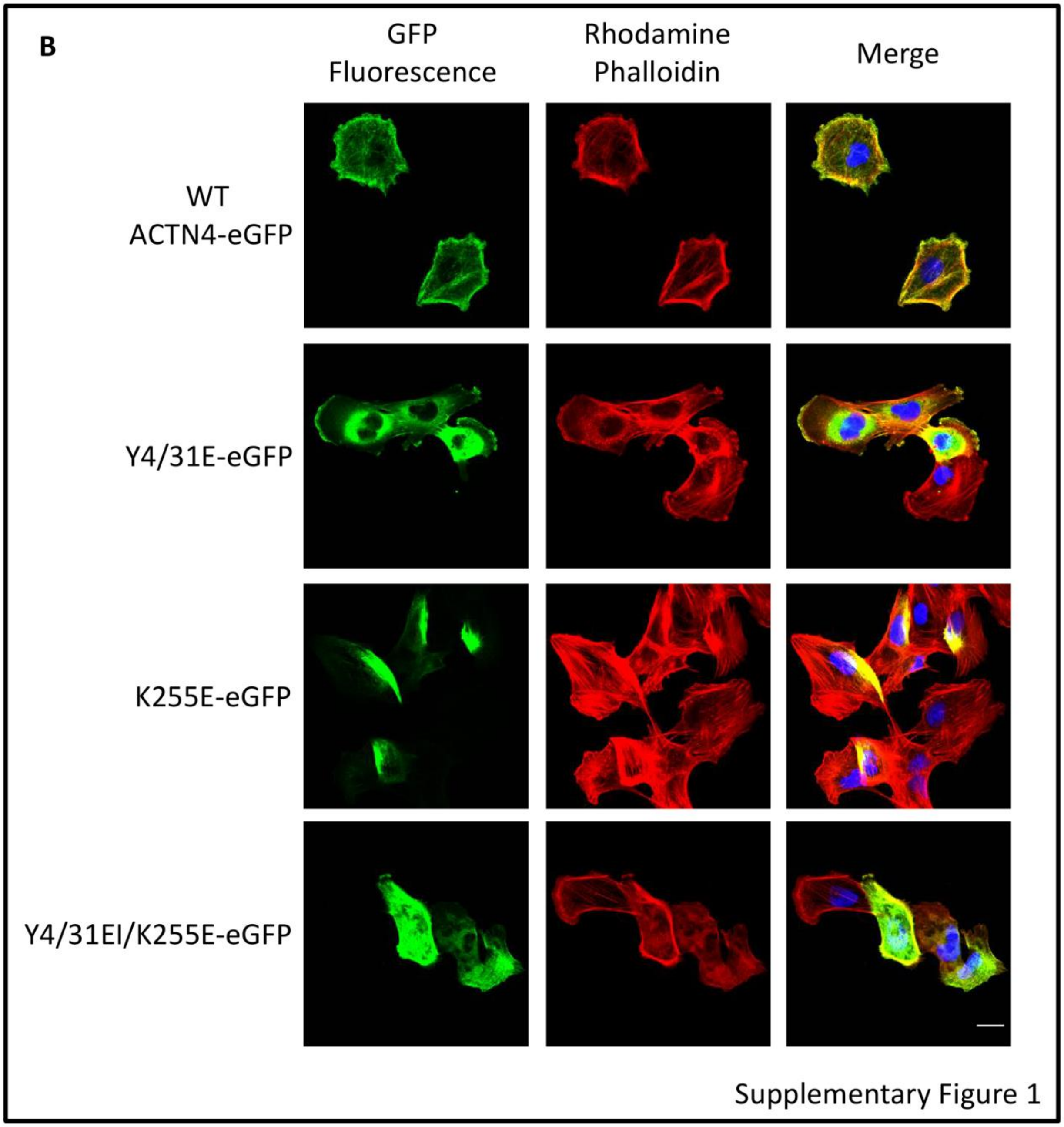

Figure S1. K255E ACTN4 does not colocalize with paxillin. **(A)** Podocytes grown in 6-well plate were transfected with indicated plasmids and then stained with paxillin antibody and DAPI. Green: fluorescence of eGFP tagged ACTN4; Red: paxillin; Blue: DAPI. **(B)** Podocytes grown in 6-well plate were transfected with indicated plasmids and then stained with rhodamine phalloidin (Red) and DAPI. Images were taken using 60x magnifications. *Scale bar*, 20 μm.

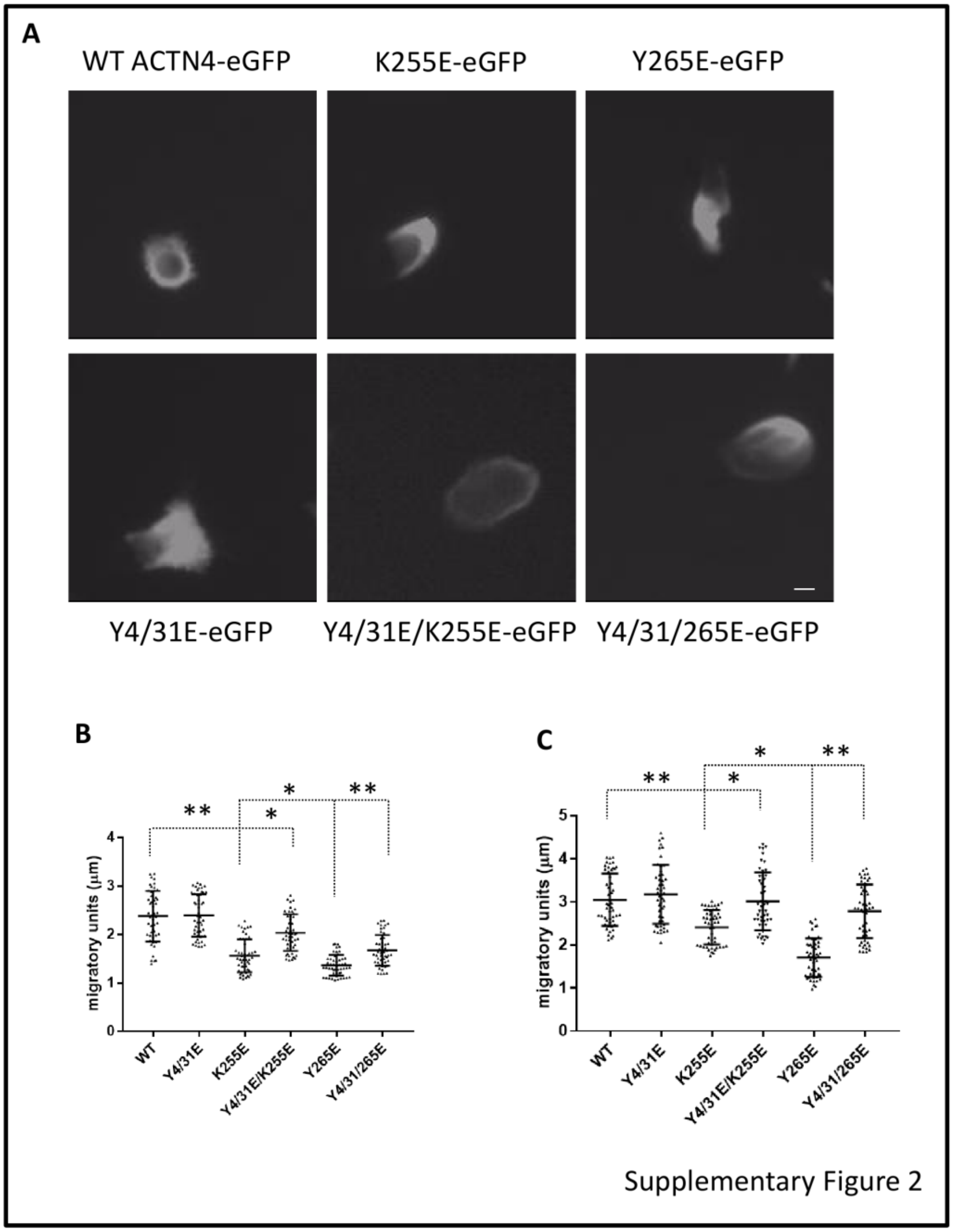

Figure S2. Y265E ACTN4 forms tight clusters during cell migration. **(A)** Representative movies of individual live migratory melanoma WM1158 cells transiently expressing GFP tagged WT or indicated ACTN4 mutants. Cells were tracked for 6 h with an interval of 10 min. Movies were created using Image J software. **(B, C)** Cell migration speed of NR6WT fibroblasts (B) and melanoma WM1158 cells (C) transiently expressing GFP tagged WT and ACTN4 mutants tracked using live microscopy and analyzed using MetaMorph software. The migratory units stand for the average distance of cells moved within 10 minutes. Cell number (N)=50. Images are a representative of three independent experiments. Data are mean of ± SD of three independent experiments. Statistical analysis was performed using Student's t-test. * $p < 0.1$, ** $p < 0.01$.

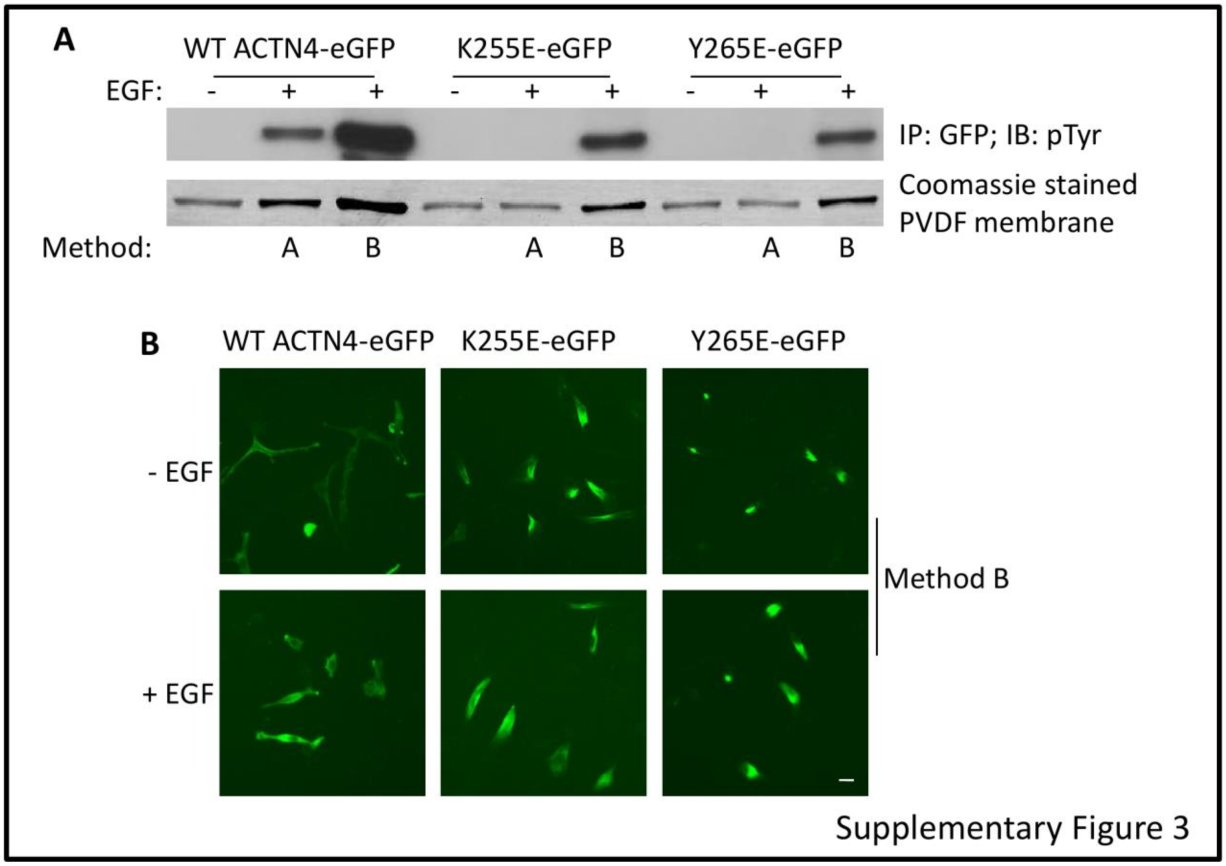

Figure S3. Continuous EGF stimulation alters the localization of both K255E and Y265E ACTN4 via phosphorylation**. (a)** Immunoblotting of immunoprecipitated GFP tagged WT, K255E and Y265E from NR6WT cells treated with EGF using method A or B. **(b)** Localization of GFP tagged WT, K255E and Y265E in NR6WT cells treated with EGF using method B. Shown are representative of three independent experiments. *Scale bar*, 20 µm.

Figure S4. Original full blots of the cropped ones in the figures.

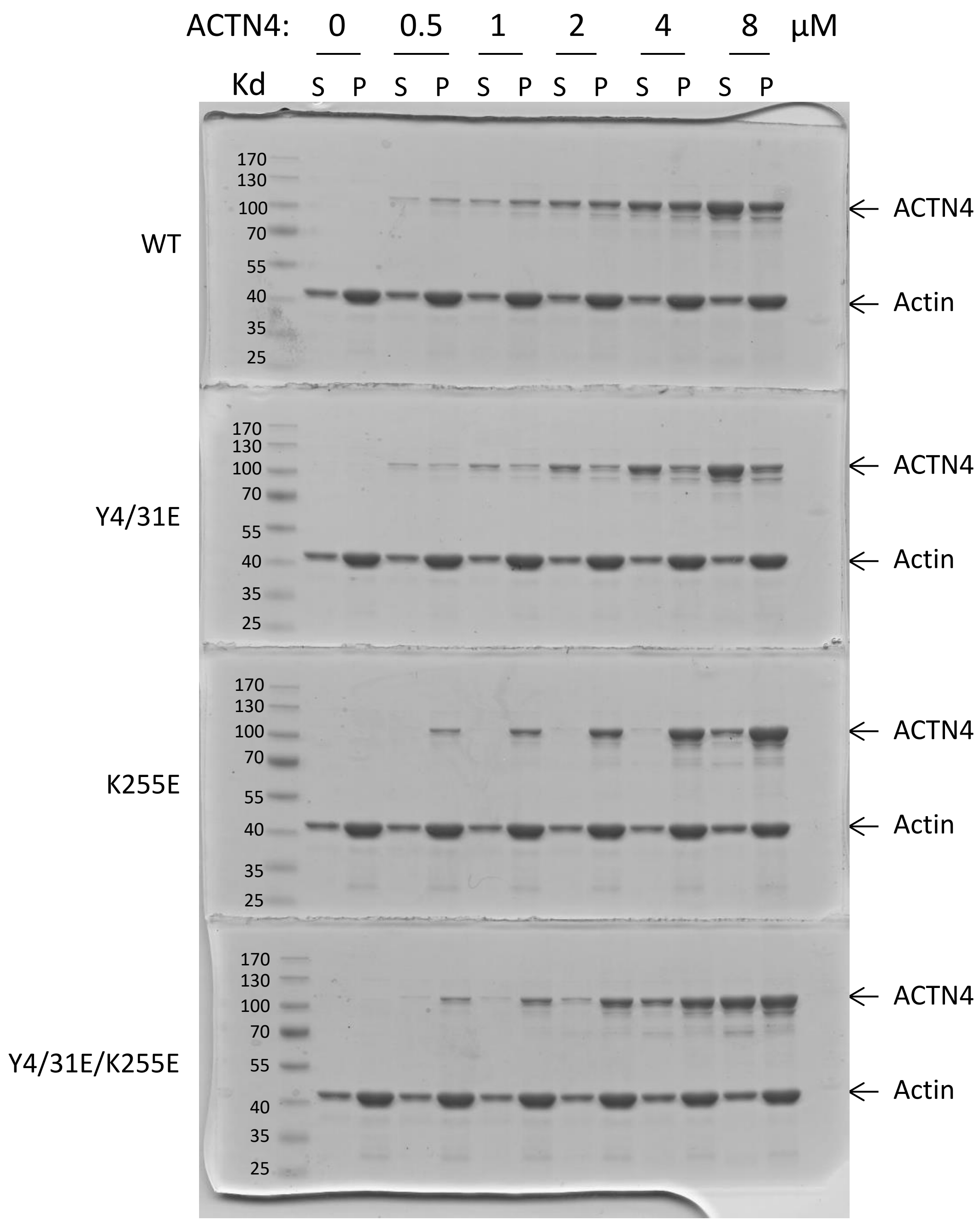

1B

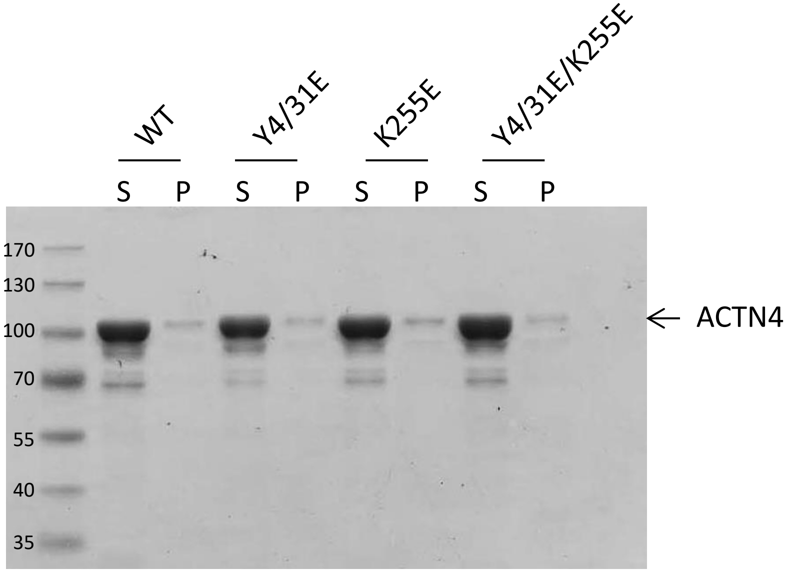

1C

Figure 1

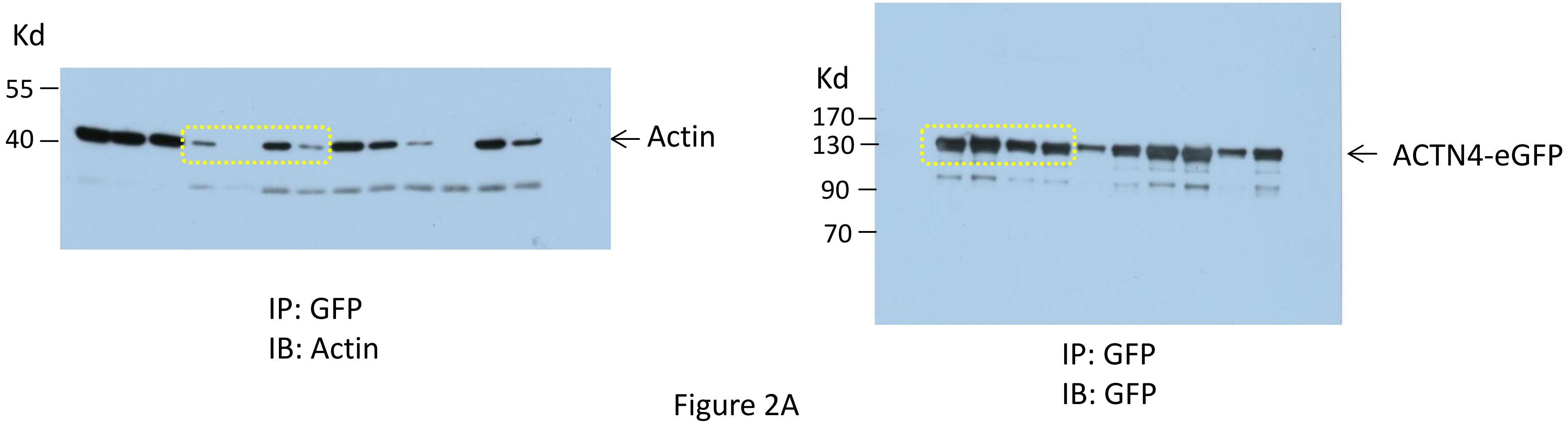

Figure 2A

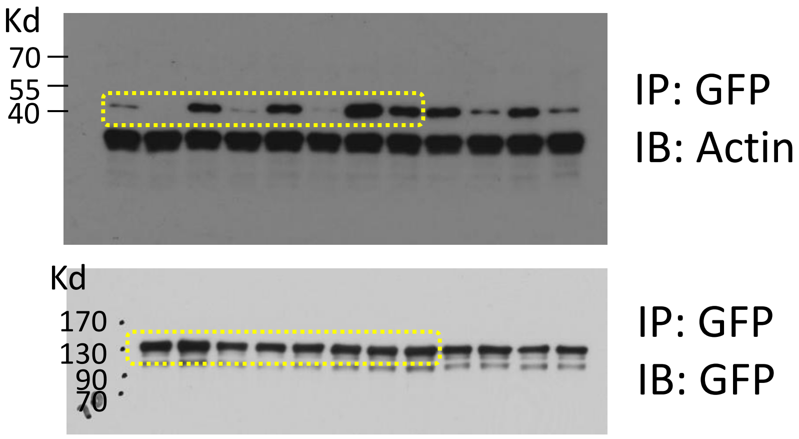

Figure 2B

Figure 2

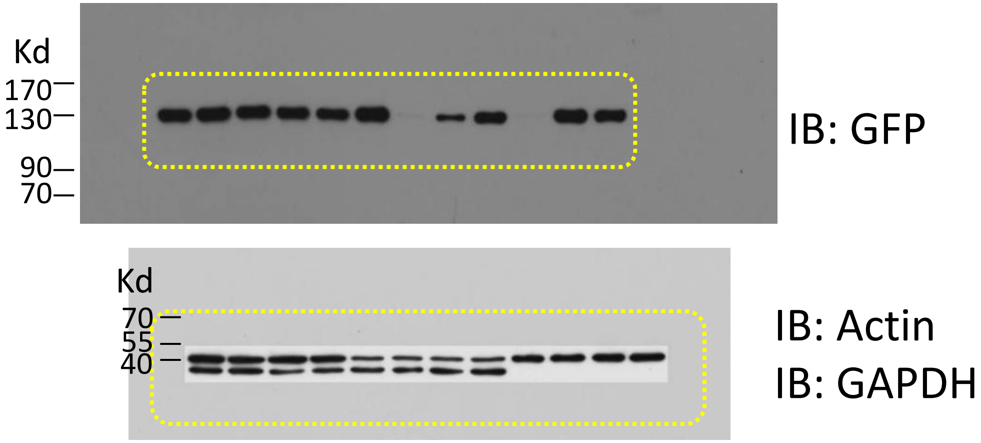

Figure 3D

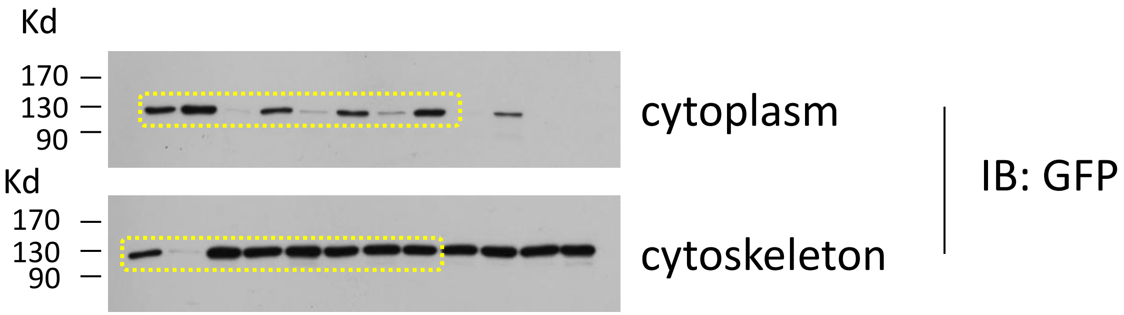

Figure 3E

Figure 3

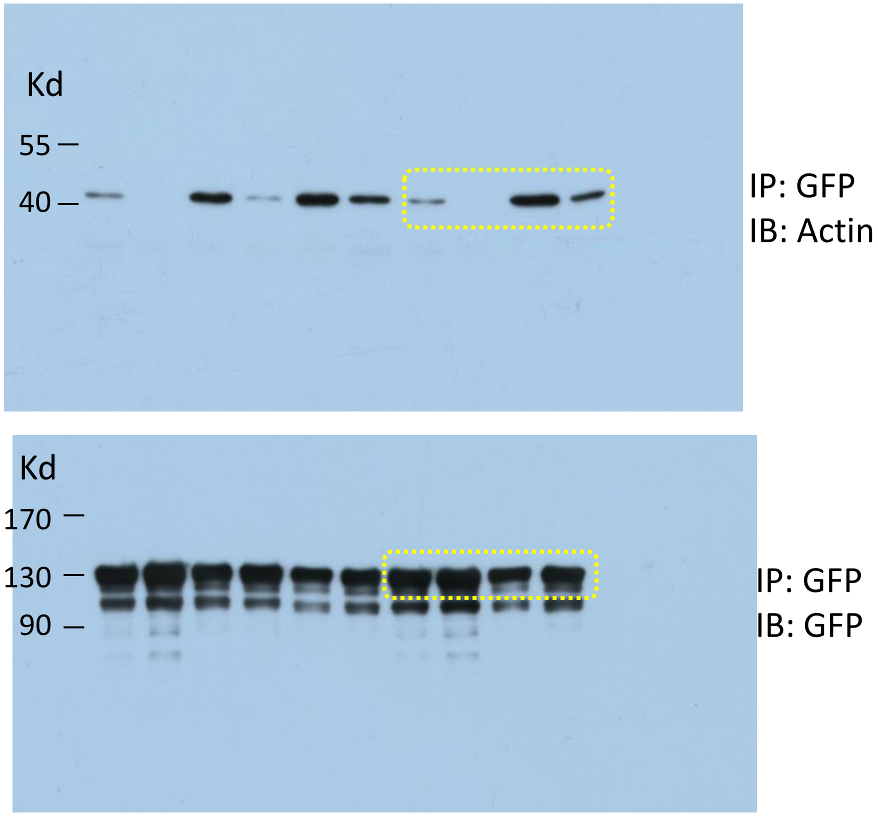

Figure 5A

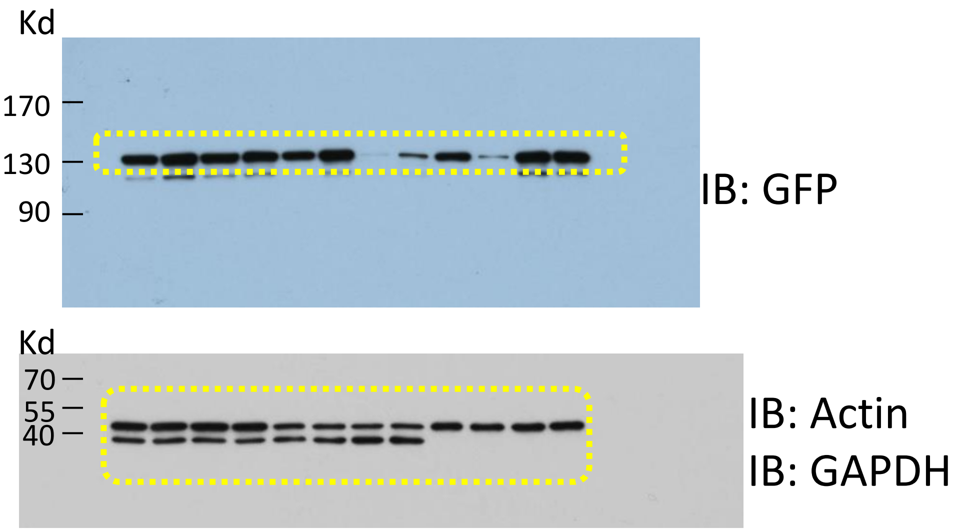

Figure 5B

Figure 5

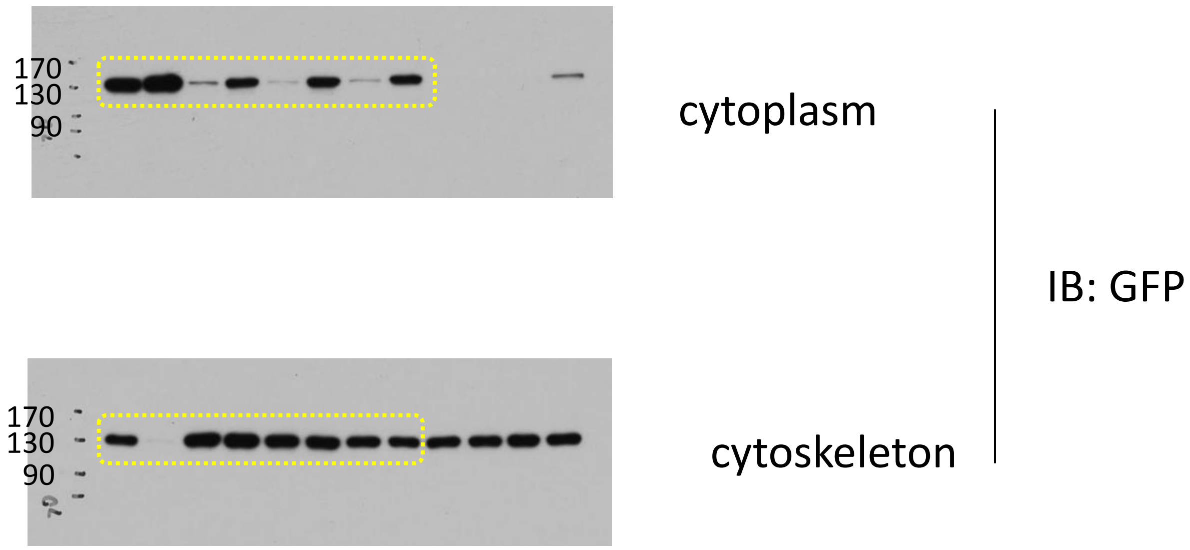

Figure 6A

Figure 6

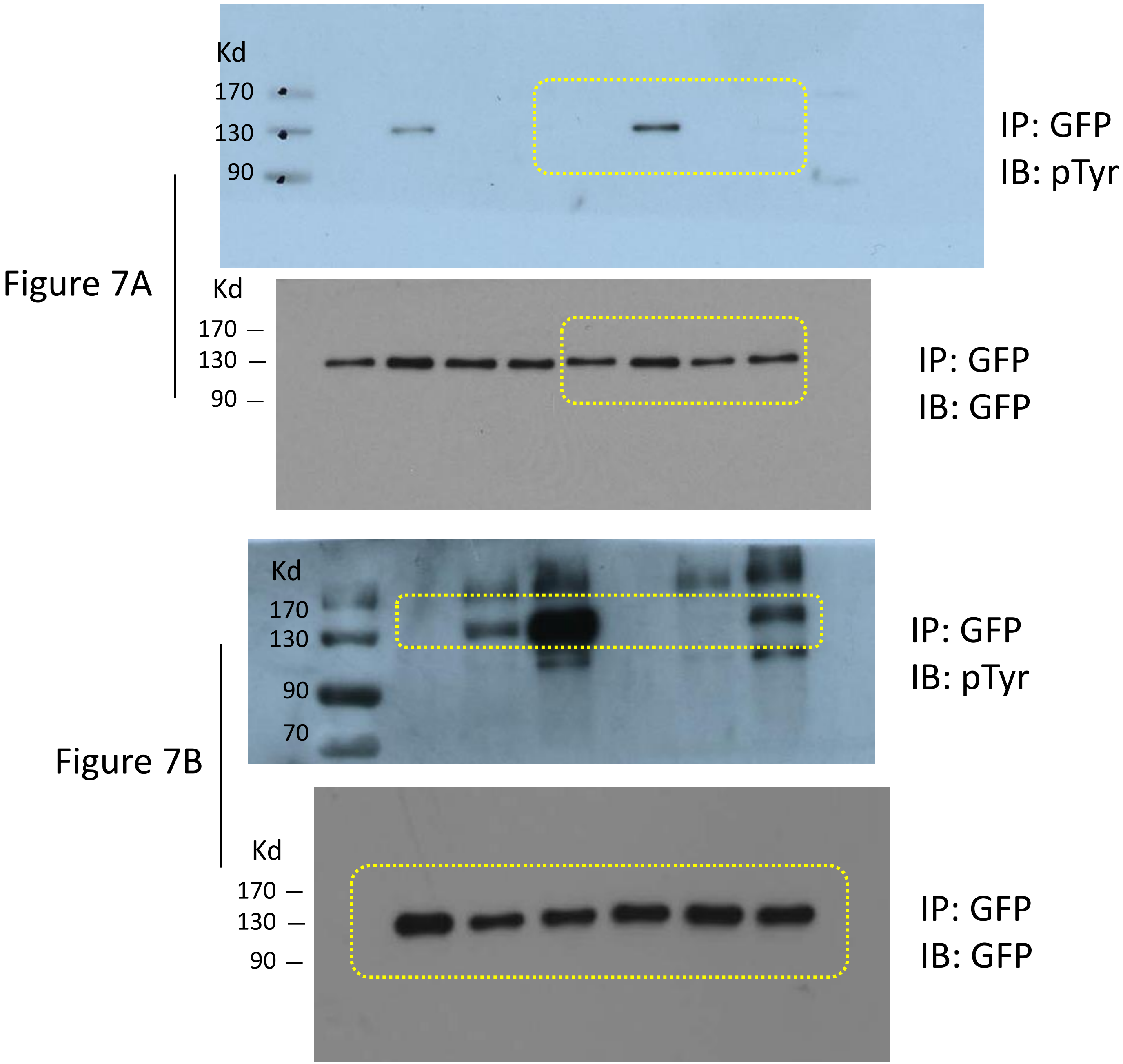
Figure 7A
Kd
170
130
90
IP: GFP
IB: pTyr
Kd
170
130
90
IP: GFP
IB: GFP
Figure 7B
Kd
170
130
90
70
IP: GFP
IB: pTyr
Kd
170
130
90
IP: GFP
IB: GFP

Figure 7

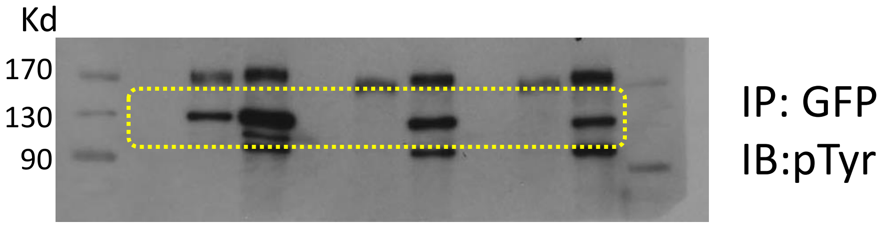

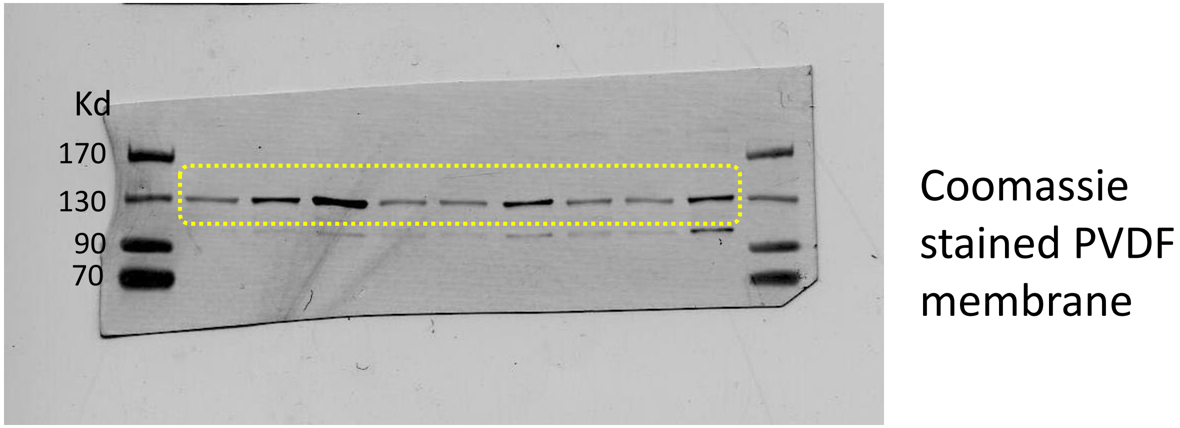

Supplemental Figure 3A
